# Symbolic Processing Mediates the Relation Between Non-symbolic Processing and Later Arithmetic Performance

**DOI:** 10.3389/fpsyg.2020.00549

**Published:** 2020-03-26

**Authors:** Sabrina Finke, H. Harald Freudenthaler, Karin Landerl

**Affiliations:** ^1^Institute of Psychology, University of Graz, Graz, Austria; ^2^Department of Cognitive Science, Macquarie University, Sydney, NSW, Australia

**Keywords:** numerical cognition, non-symbolic, symbolic, longitudinal, mediation

## Abstract

The nature of the relation between non-symbolic and symbolic magnitude processing in the prediction of arithmetic remains a hotly debated subject. This longitudinal study examined whether the influence of non-symbolic magnitude processing on arithmetic is mediated by symbolic processing skills. A sample of 130 children with age-adequate (*N* = 73) or below-average (*N* = 57) achievement in early arithmetic was followed from the end of Grade 1 (mean age: 86.9 months) through the beginning of Grade 4. Symbolic comparison of one- and two-digit numbers serially mediated the effect of non-symbolic comparison on later arithmetic. These results support a developmental model in which non-symbolic processing provides a scaffold for single-digit processing, which in turn influences multi-digit processing and arithmetic. In conclusion, both non-symbolic and symbolic processing play an important role in the development of arithmetic during primary school and might be valuable long-term indicators for the early identification of children at risk for low achievement in arithmetic.

## Introduction

The development of arithmetic skills in primary school is of fundamental importance in modern-day societies: already at the age of seven, arithmetic abilities predict adult socio-economic status over and above the effects of intelligence and socio-economic status at birth (Ritchie and Bates, 2013). Severe deficits in arithmetic are relatively stable: almost half of the children diagnosed with developmental dyscalculia at an age of 11 still meet the diagnostic criteria 6 years later (Shalev et al., 2005). Therefore, it is important to discover the cognitive mechanisms underlying arithmetic achievement in order to identify and support children at risk before their problems get persistent. However, longitudinal studies unraveling the effects of different, interacting predictors of the development of arithmetic are still scarce (Alcock et al., 2016).

Children’s arithmetic development has often been linked to their “number sense,” meaning the ability to deal with non-symbolic magnitudes, for example dots or other concrete objects. Typical tasks involve choosing the numerically larger of two sets of objects (e.g., •• or •••). This ability has been proposed to reflect the acuity of the supposedly innate approximate number system (ANS). Using a habituation-dishabituation methodology, it became apparent that 6-month-olds can differentiate between sets with a ratio of 1:2 (Xu and Spelke, 2000). During child development, non-symbolic skills are steadily refined, until young adults can successfully discriminate between sets with a ratio of 10:11 (Halberda and Feigenson, 2008).

It has been argued that non-symbolic magnitude processing is directly and causally related to arithmetic performance (Dehaene, 2002; Halberda et al., 2008). Support for this claim is mostly derived from correlational studies showing that non-symbolic processing skills are related to past, concurrent or future arithmetic performance (Halberda et al., 2008; Gilmore et al., 2010; Libertus et al., 2011). Additionally, it has been proposed that developmental dyscalculia is the result of an inborn “core deficit” of acuity of non-symbolic processing (Wilson and Dehaene, 2010).

Others have rejected the notion of a causal relation between non-symbolic magnitude processing and arithmetic, alternatively proposing that the ability to deal with abstract symbolic numbers (mainly in the form of digits) is more important for arithmetic performance. Symbolic magnitude processing is often assessed with tasks requiring participants to indicate which of two Arabic digits is numerically larger (e.g., 2 or 3). In this vein, compared to typical development, children with dyscalculia showed lower performance in a symbolic magnitude comparison task, but not when comparing non-symbolic numerosities (Rousselle and Noël, 2007). Based on a systematic review, De Smedt et al. (2013) concluded that symbolic processing is a more robust predictor of arithmetic than non-symbolic processing, as many studies failed to find a significant correlation between non-symbolic magnitude comparison and arithmetic. This assumption was recently confirmed by two meta-analyses (Fazio et al., 2014; Schneider et al., 2017) reporting a significantly stronger association with mathematics for symbolic than for non-symbolic magnitude processing. In studies that assess both, symbolic and non-symbolic processing, the latter typically does not contribute additional variance to the prediction of arithmetic over and above symbolic processing (Lyons and Ansari, 2015) leading some researchers to the conclusion that non-symbolic processing skills are “not particularly critical for children’s development of school-relevant mathematical competencies” (De Smedt et al., 2013, p. 54).

The fact that non-symbolic processing skills do not explain additional variance in arithmetic performance when controlling for differences in symbolic processing does not dismiss a potentially causal relation between non-symbolic processing and arithmetic. Only recently, an alternative mediation hypothesis has proposed that the relation between non-symbolic processing skills and arithmetic might be mediated by symbolic skills (Lyons and Beilock, 2011; Fazio et al., 2014; van Marle et al., 2014; Price and Fuchs, 2016; Peng et al., 2017; Träff et al., 2018). An evolutionary based ability to discriminate between sets of objects may provide a starting point for young children’s mapping of numerical symbols (number words, Arabic numbers) onto non-symbolic numerosities, which in turn are the foundation of their arithmetic skills (Dehaene, 2002). Nevertheless, empirical support for the claim that non-symbolic skills provide a scaffold for symbolic skills, which in turn predict arithmetic performance, is mostly based on cross-sectional studies.

For instance, Lyons and Beilock (2011) found that ordering skills fully mediated the association between non-symbolic processing skills and arithmetic in a sample of young adults. The authors argued that the ability to comprehend the relative order of digits might be grounded in an ANS and act as a stepping stone for the acquisition of arithmetic skills in a small sample (*N* = 53) of fifth graders, Fazio et al. (2014) found that composite scores of non-symbolic and symbolic processing independently contributed to the prediction of mathematics. When the authors tested for a possible indirect effect by examining the reduction of the direct effect of non-symbolic skills on arithmetic performance once symbolic processing was added. This reduction of the direct effect just about missed significance. Thus, there is at least some evidence for a weak indirect effect which might have well been significant if the sample size had been larger.

If non-symbolic processing is a foundation of understanding symbolic numbers, it might be expected that it is of particular relevance in young children who are still developing their symbolic number system. Indeed, Peng et al. (2017) reported for a sample of kindergarten children aged five to six that a composite “numerical knowledge” variable significantly mediated the relation between non-symbolic processing skills and arithmetic, even when controlling for a variety of covariates, including intelligence, working memory, attention and inhibition. Numerical knowledge consisted of rapid automatized naming with digits, identification of one- to three-digit numbers, and numerical reasoning (completing a sequence of numbers). Similarly, Price and Fuchs (2016) found full concurrent mediation of the relation between non-symbolic processing and arithmetic by symbolic processing in a sample of 9-year-olds, even when working memory skills were controlled for. In order to keep task requirements as similar as possible, non-symbolic and symbolic skills were both assessed by comparison tasks encompassing numerosities from 1 to 9. Importantly, non-symbolic processing did not conversely mediate the effect of symbolic processing on arithmetic. In a similar age group of third graders, Träff et al. (2018) also found evidence for an indirect effect of non-symbolic processing speed on single-digit arithmetic mediated by symbolic processing speed, over and above the influence of linguistic skills, as indexed by language comprehension and rapid automatized naming. Non-symbolic processing skills were measured with a computerized task comprising numerosities between 5 and 21 per array (Panamath; Halberda et al., 2008), whereas the symbolic processing measure consisted of a composite score of single- and double-digit comparisons. In another study with 5- to 8-year-olds (Li et al., 2018) non-symbolic as well as symbolic processing tasks were assessed in numerosities from 5 to 50. Interestingly, the effect of non-symbolic processing on mathematical ability was mediated by symbolic processing skills in children aged 5–6, but not in 7- to 8-year-olds, providing first evidence that the age of assessment may be critical.

Longitudinal studies are particularly relevant in order to determine causal mechanisms during development. So far, only one such study (van Marle et al., 2014) investigated symbolic skills as a potential mediator of the relation between non-symbolic magnitude processing in 3- to 4-year-olds on entering preschool and mathematic abilities at the end of the preschool year. The mathematical abilities test encompassed items involving enumeration, counting, cardinal knowledge and numeral identification, but critically, calculation skills or arithmetic fact knowledge could not be assessed in this young age group, which may well explain why symbolic magnitude comparison was not found to be a mediator for the relation between non-symbolic processing and these very basic mathematical competences.

In summary, several studies investigating the hypothesis that symbolic processing abilities serve as a mediator of the relation between non-symbolic processing skills and arithmetic did indeed report some evidence in favor of this claim. Conflicting findings might in part be due to the different measures of symbolic number processing that were employed (e.g., ordinality judgment, numerical recognition, and number comparison) and differences in age groups assessed. Still, the foundational link between non-symbolic and symbolic processing is not entirely uncontested: while there is some evidence that non-symbolic processing influences the development of symbolic processing skills (Toll et al., 2015), other studies could not corroborate this link and reported that, on the contrary, symbolic skills predicted growth in non-symbolic processing (Mussolin et al., 2014; Matejko and Ansari, 2016; Lyons et al., 2018). Therefore, it appears crucial that studies testing a mediation model of non-symbolic processing on arithmetic via symbolic processing should control for initial symbolic skills, in order to test whether non-symbolic processing actually predicts growth in symbolic processing skills.

In addition, it might also be important to differentiate between distinct subcomponents of symbolic processing, in particular single- and multi-digit number processing. There is increasing evidence suggesting that multi-digit number processing differs from single-digit processing and is acquired later in development (Brankaer et al., 2017). Moreover, it has been proposed that single-digit number processing constitutes a necessary first step, while additional specific processes, such as place-value knowledge, are required to fully understand multi-digit numbers (Nuerk et al., 2015). Thus, it seems plausible to assume that the ability to process multi-digit numbers is scaffolded onto single-digit number processing, which in turn may rely on non-symbolic processing.

In the current study we tested a developmental model of sequential mediation of the effect of non-symbolic processing on later arithmetic performance via processing of one- and two-digit numbers. This developmental account was investigated in a longitudinal study ranging from end of Grade 1 to beginning of Grade 4. In this important period of arithmetic development, children are introduced to the complexities of the Arabic place-value system and are expected to acquire fluent competencies in mental calculation and to store a large amount of easily accessible number facts in their long-term memory.

Non-symbolic, single- and multi-digit number processing were assessed at different, sequential time points, and prior to arithmetic skills. When testing our developmental framework, we controlled for general cognitive skills that have been found to be associated with arithmetic performance and might influence its relation with non-symbolic processing, i.e., non-verbal IQ (Göbel et al., 2014b), verbal working memory (Berg, 2008), and attention/executive functions (Clark et al., 2013). Furthermore, as the children had already gained substantial experience with symbolic numbers and arithmetic at the beginning of our study period, it was important to additionally control for initial symbolic and arithmetic skills.

Based on the findings of previous studies, we expect that non-symbolic processing exerts an indirect effect of future arithmetic performance but may not uniquely contribute to the prediction of arithmetic performance (i.e., no total effect) when considering these control variables. Note that this study design puts the hypothesis that non-symbolic processing is a foundational skill underlying symbolic processing and arithmetic at a very stringent test: while non-symbolic processing could be expected to have its strongest influence early in development, when numbers are mapped onto analog magnitudes, our longitudinal design mainly assesses whether differences in non-symbolic processing contribute to growth of symbolic processing and (in turn) arithmetic skills during the primary school years, over and above general cognitive predictors of arithmetic. If any such indirect long-term effects can be demonstrated, even though small, the hypothesis that the non-symbolic magnitude processing is a foundational skill of arithmetic should be further investigated.

## Materials and Methods

### Participants

The study was conducted in accordance with the ethical principles of the World Medical Association Declaration of Helsinki. Data collection started in 2007 and at that time the funding agency (DFG) and local legislation did not request an explicit vote from an ethics committee for non-medical research. Legal guardians gave their written informed consent before data collection. The present sample consisted of 130 children from 19 different elementary schools and a total of 38 classrooms taking part in a longitudinal study investigating the developmental trajectories of basic numerical skills in children with typical and atypical arithmetic development (Landerl, 2013).

The participants were invited to the study based on a screening of 505 children at the end of first grade. Children with arithmetic achievement of 1 *SD* or more below age norm on a standardized test (Haffner et al., 2005) were all invited for additional assessments. For each participant with below-average arithmetic achievement, we selected one child from the same classroom who displayed typical arithmetic development (i.e., arithmetic performance above −1 *SD* compared to the age norm). Thus, children with low arithmetic performance in Grade 1 were overselected in our sample. As our focus was on numerical and arithmetic development, we attempted to exclude more general deficits in non-verbal IQ, working memory, attention, and reading as potential causes or confounds of arithmetic deficits. More specifically, children were not admitted to the study if they met any of the following exclusion criteria:

• a native language other than German;
• IQ lower than 85 as assessed by a test of non-verbal intelligence (Cattell et al., 1997);
• verbal working memory more than 1 *SD* below age norm on the German version of the WISC-IV digit span subtest (Petermann and Petermann, 2008);
• a clinical diagnosis of attention deficit/hyperactivity disorder or performance more than 1.5 *SD*s below age norm on a standardized test of attention/executive functions (Zimmermann et al., 2002);
• reading abilities more than 2 *SD*s below age norm, as measured by a standardized reading test (Mayringer and Wimmer, 2003). As the deficits of children with co-occurring dyslexia and dyscalculia appear to be additive but not qualitatively different from isolated disorders (Landerl et al., 2009), a conservative cut-off for reading problems was chosen.

The initial sample consisted of 139 children (68 boys and 71 girls), of whom 131 participated through Grade 4. One child with low arithmetic performance had to be excluded because of below chance-level performance on the non-symbolic comparison task (rendering it unclear whether this child had understood the instruction). Thus, the final sample comprised 130 children (60 boys and 70 girls) with an average age of 86.9 months at the screening (end of Grade 1). At this first assessment point, 57 children showed low arithmetic performance. At the last assessment point in Grade 4, only 39 children performed more than 1 *SD* below the age norm in arithmetic, while the majority of the sample (*N* = 91) showed arithmetic skills within the typical range.

### Design

**FIGURE 1:**
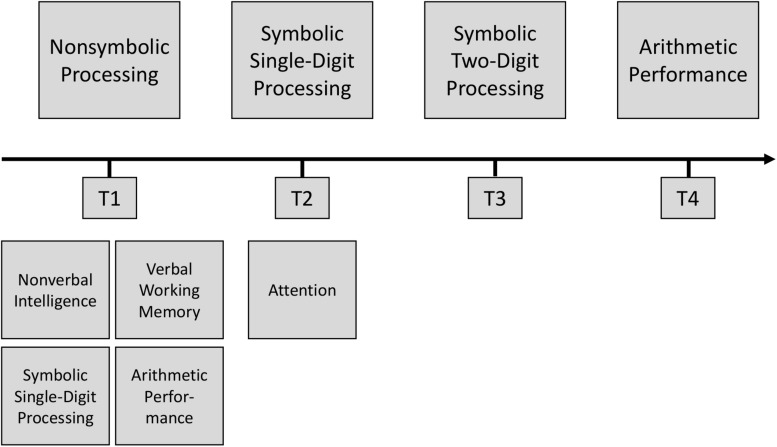
Overview of the study design. Main study variables are depicted above the timeline and covariates are depicted below the timeline.

Children’s development was followed across a 2-year primary school period from end of Grade 1 (T1) to beginning of Grade 4 (T4). The first assessment point (T1) subsumed measures that were either given at the end of Grade 1, or right at the beginning of Grade 2, interspersed only by 6 weeks school holidays. Non-symbolic processing at T1 was considered as independent predictor variable. Symbolic single-digit processing was assessed 6 months later in the middle of Grade 2 (T2) and symbolic two-digit processing was assessed after another 6 months at the beginning of Grade 3 (T3). The dependent variable arithmetic performance was assessed at the beginning of Grade 4 (T4). We additionally considered several covariates: non-verbal intelligence, attention, verbal working memory, as well as initial arithmetic performance and symbolic single-digit processing (all T1, except for attention, which was assessed at T2 because of restricted assessment time at T1). An overview of the study design is depicted in Figure 1.

### Tasks

#### Numerical Processing

Non-symbolic and symbolic processing were assessed by standard numerical comparison paradigms programmed with Presentation software. Children performed the tasks individually in a quiet room at their school. We obtained a combined measure of speed and accuracy for children’s non-symbolic and symbolic processing skills by calculating inverse efficiency scores (median reaction times divided by the proportion of correct responses).

##### Non-symbolic comparison

**FIGURE 2:**
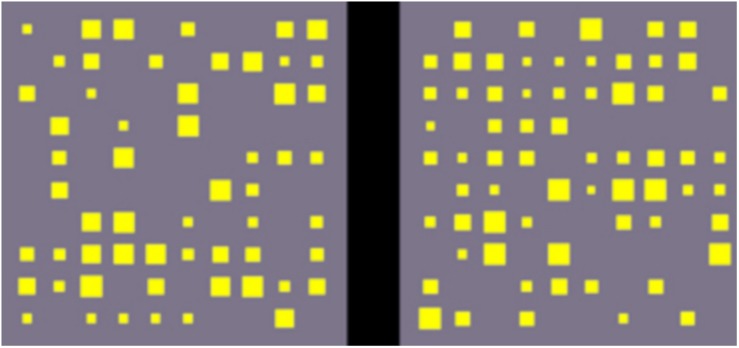
Example item of the magnitude comparison task (Landerl, 2013).

Children were required to indicate which of two gray displays had the larger number of yellow squares (see Figure 2) by pressing the corresponding keyboard button as rapidly as possible. The number of squares per display ranged from 20 to 72 squares in order to discourage children from explicit counting. The difference between displays’ set sizes ranged from 8 to 25 squares, with four trials for each numerical distance, resulting in a total of 72 test items. The total surface area was the same on both displays, and the same proportion of both displays was covered by yellow squares. Each display consisted of different square sizes to avoid that displays with larger numerosities systematically consisted of smaller squares. The largest and smallest squares appeared in the same number in both displays; only size and number of intermediate squares were different. Stimuli were displayed in a fixed pseudo-random order and remained on screen until the child made a keypress decision. After an interstimulus-interval of 300 ms, the next item appeared. At the start, three practice items were presented. Cronbach’s alpha reliability for the non-symbolic comparison task was 0.95.

##### Symbolic comparison

Two tasks assessed symbolic processing skills: single digit comparison (T1 and T2) and comparison of two-digit numbers (T3). In both tasks, children chose the numerically larger of two numbers by pressing the corresponding keyboard button as quickly as possible. The single digit comparison task consisted of 56 items with numbers from 1 to 9. Numerical distances ranged from 1 to 8 (distance 1: 16 items, distances 2–3: 10 items, and distances 4–8: 4 items). Comparison of two-digit numbers comprised 80 items with numbers between 21 and 98. Numerical distance ranged from 4 to 37. In 30 items, both decade and unit digit were larger in one number (e.g., 41 75), in 30 items, the decade digit was larger in one and the unit digit was larger in the other number (e.g., 41 26), and in further 20 items, only the unit digit differed (e.g., 61 68). In both symbolic processing tasks, stimuli were displayed in a 36-point Times New Roman font in black color against a white background. Item presentation was randomized and the number pairs remained on the screen until children responded by keypress. After each item, there was an interstimulus-interval of 560 ms. For both symbolic comparison measures, Cronbach’s alpha ranged between 0.96 and 0.97.

#### Arithmetic Performance

Arithmetic performance was assessed by the “arithmetic operations” subscale of a standardized classroom test (Haffner et al., 2005). At T1, the assessment included lists of addition, subtraction, fill-in-the-blank (e.g., 10 – 2 = 4 + _) and size comparison exercises (e.g., 51 – 1 _ 6; fill in “>”). At T4, two additional subtests targeting multiplication and division were included. Each subtest had a 2 min time limit with items being presented with increasing difficulty. Within this time limit, children were required to write down as many correct answers as possible to a list of calculations gradually increasing in difficulty. Performance in each subtest was assessed as the number of correct answers. A composite measure “arithmetic operations” was calculated as the mean of the standardized *T*-scores (mean = 50, *SD* = 10) of all subtests.

#### Non-verbal Intelligence

Non-verbal intelligence was assessed by the German version of the Culture Fair Test (Cattell et al., 1997), comprising the subtests substitutions, mazes, classifications, similarities and matrices. These five subtests provided a measure of general intellectual ability, i.e., a child’s ability to recognize regularities and quickly identify characteristics.

#### Attention

Children performed a standardized computer-based test battery encompassing different facets of attention/executive functions (Zimmermann et al., 2002). Attention was indexed by a composite score of the subtests distractibility, alertness, sustained attention, flexibility, and divided attention. In the distractibility subtest, children were required to selectively press a button upon seeing a ghost with a sad face. In half of the trials, a distractor in form of a ghost with a happy face appeared right before the target stimulus. In the alertness subtest, a witch appeared in the center of the screen at varying intervals and children had to press a button as quickly as possible. The sustained attention subtest measured children’s ability to maintain their attention over a longer period of time (10 min) by watching the color of ghosts that appeared on the screen one after the other. They had to press a button whenever two subsequent ghosts had the same color. In the flexibility subtest, a green and a blue dragon appeared simultaneously on the screen, and children had to indicate the positions of both dragons. In alternating trials, the position of the green versus the blue dragon had to be indicated first. During the divided attention subtest, children were presented with different visual and auditory stimuli. They had to react to changes in the stimuli, i.e., when an owl closed its eyes or changed its hooting.

#### Verbal Working Memory

The Digit Span subtest (forward and backward combined) of the German version of the Wechsler Intelligence Scale for Children IV (Petermann and Petermann, 2008) was used to assess verbal working memory. In the forward condition, children were required to repeat a string of verbal numbers presented by the experimenter in the same order. In the backward condition, they were asked to repeat the number strings in the inverse order. For each number length, two items were presented and a discontinuation rule applied if a child was unable to repeat at least one of these items. Verbal working memory was indexed as the total score of correctly recalled number strings.

## Results

For each of the numerical processing tasks, individual median response times were calculated after removing reaction times for incorrect responses, below 200 ms and above 10,000 ms. In the non-symbolic comparison task the correlation between median RTs and response accuracy was only moderate, *r* = 0.324, *p* < 0.001 and response accuracy was close to ceiling in both symbolic comparison tasks (mean accuracy symbolic comparison with single-digit numbers at T1 = 95.5% and T2 = 96.6% and symbolic comparison with two-digit numbers at T3 = 91.3%). In order to combine response accuracy and speed, inverse efficiency scores were computed for the non-symbolic and symbolic tasks by dividing the median reaction time by the proportion of correct responses. Finally, one extreme outlier score in the non-symbolic comparison task (more than 6 *SD*s above the sample mean) was moved to the tail of the distribution to the second highest score to avoid overemphasizing its effect on the results.

### Descriptive Statistics

Means, standard deviations and intercorrelations of all relevant study variables are shown in Table 1. Pearson correlation coefficients were reported to describe the linear relations between study variables. If a pair of those variables was not bivariately normally distributed, confidence intervals obtained by bootstrapping and Spearman coefficients were also computed to examine statistical significance. In all of these cases, the three approaches yielded identical results regarding statistical significance.

**TABLE 1:** Descriptive statistics and correlations between all relevant study variables.

Variable	Mean	*SD*	1	2	3	4	5	6	7	8	9
(1) Arithmetic T1^a^	45.50	9.11	–								
(2) Non-symbolic T1^b^	1686.16	353.41	−0.29**	–							
(3) Non-verbal Intelligence T1^c^	108.25	11.71	0.49**	−0.22*	–						
(4) Symbolic Single-Digit T1^b^	1103.76	247.56	−0.41**	0.47**	−0.24**	–					
(5) Verbal Working Memory T1^b^	11.80	1.82	0.31**	–0.06	0.18*	–0.06	–				
(6) Attention T2^a^	47.03	4.46	0.46**	−0.31**	0.39**	−0.45**	0.20*	–			
(7) Symbolic Single-Digit T2^b^	985.04	216.75	−0.42**	0.47**	−0.36**	0.69**	0.01	−0.38**	–		
(8) Symbolic Two-Digit T3^b^	45.50	9.11	−0.43**	0.46**	−0.35**	0.62**	–0.08	−0.33**	0.62**	–	
(9) Arithmetic T4^a^	1686.16	353.41	0.72**	−0.27**	0.51**	−0.40**	0.22*	0.41**	−0.48**	−0.50**	–

^a^Mean T-Score of all Subtests (M:50/SD:10).^b^Inverse Efficiency Score. ^c^IQ Score (M:100/SD:15). * p < 0.05; ** p < 0.01.

### Mediation Analyses

Mediation analyses were calculated using the PROCESS macro for SPSS (Hayes, 2013). In order to evaluate the hypothesis that the influence of non-symbolic magnitude processing on later arithmetic performance is sequentially mediated by symbolic magnitude processing of single- and multi-digit numbers, we calculated a serial multiple mediation analysis with bootstrapping. During the bootstrapping procedure, the current sample was randomly resampled with replacement. An empirically obtained representation of the sampling distribution of the indirect effect was used to generate the confidence interval for the indirect effect. In the current study, we employed a bias-corrected bootstrap with a 95% confidence intervals based on 10,000 bootstrap samples.

**FIGURE 3:**
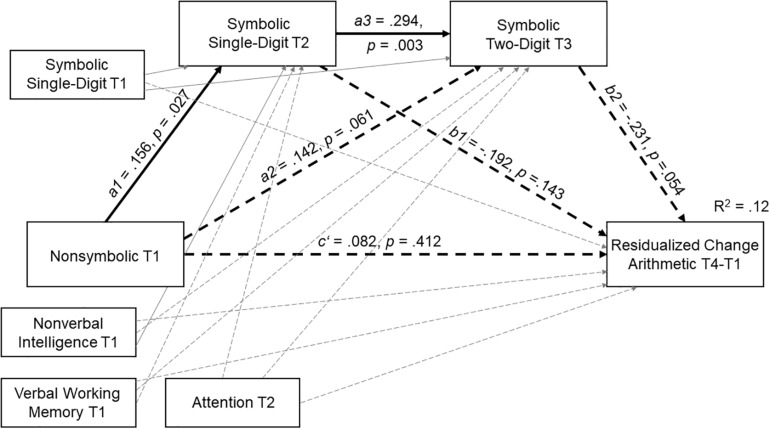
Serial multiple mediation model for the effect of non-symbolic processing on residualized change scores in arithmetic performance T4-T1 with symbolic processing of single- and two-digit numbers as mediators.

Residualized change scores of arithmetic (arithmetic T4 – arithmetic T1) were considered as dependent variable, and symbolic processing of single- and two-digit numbers were introduced as mediators, so we obtained the following PROCESS model: non-symbolic processing T1 → symbolic processing (single-digit numbers) T2 → symbolic processing (two-digit numbers) T3 → residualized change in arithmetic T4-T1. We added several general cognitive covariates of both mediators and the dependent variable, namely verbal working memory, non-verbal intelligence and attention. We also included a fourth covariate, symbolic processing of single-digit numbers at T1. The full PROCESS model including the main standardized path coefficients is depicted in Figure 3. The only significant direct effects were from non-symbolic to symbolic single-digit processing (*a1* path) and from single-digit to two-digit processing (*a3* path). Importantly, neither non-symbolic processing skills at T1 nor symbolic single-digit processing at T2 exerted significant direct effects on arithmetic growth (*c*′ and *b1* paths). The direct paths from non-symbolic to two-digit symbolic processing (*a2*) and from two-digit symbolic processing to arithmetic growth (*b2*) missed significance (*p*s = 0.061 and 0.054, respectively).

Indirect effects, their standard errors and confidence intervals are presented in Table 2. Non-symbolic processing did not exert a significant indirect effect on arithmetic growth through symbolic processing of single-digit numbers at T2 (*a1b1* path) or through symbolic processing of two-digit numbers at T3 (*a2b2* path). Still, non-symbolic processing did show a significant (though small) influence on arithmetic growth serially through symbolic processing of single-digit numbers at T2 and symbolic processing of two-digit numbers at T3 (*a1a3b2* path).

**TABLE 2:** Effects, standard errors, and bootstrapped confidence intervals of non-symbolic processing on residualized change scores in arithmetic between T1 and T4 (controlling for non-verbal intelligence, attention, verbal working memory, and symbolic magnitude processing at T1, contributing to the mediators and arithmetic performance).

Effects			Estimate (*SE*)	LCI	UCI
Direct:	*c*′	Non-symbolic T1 → Arithmetic Growth T4 – T1	0.082 (0.100)	−0.112	0.280
Indirect:	*a1b1*	Non-symbolic T1 → Symbolic Single-Digit T2 → Arithmetic Growth T4 – T1	−0.030 (0.027)	−0.113	0.003
	*a1a3b2*	Non-symbolic T1 → Symbolic Single-Digit T2 → Symbolic Two-Digit T3 → Arithmetic Growth T4 – T1	−0.011 (0.011)	−0.057	−0.001
	*a2b2*	Non-symbolic T1 → Symbolic Two-Digit T3 → Arithmetic Growth T4 – T1	−0.033 (0.032)	−0.135	0.005
Total:		Non-symbolic T1 Arithmetic Growth T4 – T1	0.008 (0.099)	−0.118	0.205

*LCI, lower CI bound; UCI, upper CI bound.*

## Discussion

### Non-symbolic Magnitude Processing and Arithmetic

The presented sequential mediation analyses indicated that the effect of non-symbolic processing in Grade 2 on arithmetic performance 2 years later, in Grade 4, was sequentially mediated by symbolic magnitude processing of one- and two-digit numbers. Even though this mediation effect was small, these results provide an important empirical contribution to the ongoing debate whether non-symbolic processing skills make a causal contribution to arithmetic development. Our developmental perspective on the association of magnitude processing with arithmetic (see also Verguts and Fias, 2004; von Aster and Shalev, 2007) is consistent with evidence that symbolic processing is more strongly associated with arithmetic than non-symbolic processing (De Smedt et al., 2013; Schneider et al., 2017). The fact that the contribution of non-symbolic processing to later arithmetic is indirect via symbolic processing skills can explain why non-symbolic processing did not account for variance above and beyond symbolic processing in earlier studies (Göbel et al., 2014b; Lyons and Ansari, 2015). Our results support the theoretical view that early non-symbolic processing skills make a small but significant contribution to later arithmetic performance. Importantly however, this contribution is completely indirect by providing a scaffold for symbolic processing of one- and two-digit numbers.

Regarding the hypothesis that non-symbolic processing influences arithmetic via symbolic processing, the current longitudinal evidence provides support for the causal claims made based on cross-sectional studies (Price and Fuchs, 2016; Peng et al., 2017; Träff et al., 2018). By covering a relatively long period of over two critical years of early mathematical development and considering a variety of possible confounding factors, we extended the findings by van Marle et al. (2014) on their kindergarten sample. We found a significant direct contribution to symbolic processing of single-digit numbers and an almost significant direct contribution to symbolic processing of double-digit numbers. Importantly, we found a significant long-term indirect contribution of non-symbolic processing skills to arithmetic growth toward the end of the primary school period. A further distinctive feature of our design was that we controlled for early differences in non-verbal IQ, verbal working memory and attention. As expected and consistent with earlier research (Berg, 2008; Clark et al., 2013; Göbel et al., 2014b), these general cognitive factors were significantly related to growth in arithmetic skills across the study period. It is particularly impressive that the mediation pathway from early non-symbolic magnitude processing to growth in arithmetic was significant across these critical years of primary school and beyond the influence of these general cognitive predictors and even after controlling for interindividual differences in single-digit symbolic processing at the onset of the study period. Given the design of our study, it is not particularly surprising that this effect was numerically small and one could argue that it is irrelevant as its ecological validity is low. However, from a theoretical point of view, such a small but significant long-term effect suggests that current proposals that non-symbolic processing may be entirely irrelevant for understanding symbolic representations of number and arithmetic (De Smedt et al., 2013) are perhaps premature. On the contrary, our findings encourage further research on the exact mechanisms underlying the associations of non-symbolic processing with symbolic numerical processing and arithmetic skills from a developmental perspective.

### Non-symbolic and Symbolic Processing

In contrast to a number of recent studies, we found a significant contribution of early non-symbolic processing to growth in symbolic processing skills half a year later even after controlling for a variety of general cognitive variables. A number of other longitudinal studies with kindergarten and first grade children failed to find a similar contribution (Mussolin et al., 2014; Matejko and Ansari, 2016; Lyons et al., 2018). It is not unlikely that the special characteristics of the sample investigated here increased the chance to reveal such a relation. At the onset of the study, almost half of our participants were selected because they showed early problems in arithmetic performance. Therefore, the variance in non-symbolic and symbolic processing skills was perhaps larger than in randomly selected samples, which may have helped to reveal a relation that is small and therefore hard to detect in the normal population. It is also possible that this association is only evident in individuals with deficits in arithmetic development. This would be in line with assumptions that there may be two subtypes of dyscalculia: one with a core deficit in non-symbolic magnitude processing and another one with intact magnitude processing but problems to access magnitude representations from symbolic number representations (Rousselle and Noël, 2007). Depending of the profiles of individual participants within a sample, findings may vary. Unfortunately, our sample was too small to run separate analyses for children with arithmetic deficits and as a matter of fact, only a subgroup of those with early problems turned out to develop persistent deficits in arithmetic. In future studies, it might be worthwhile to investigate whether the early relation between non-symbolic processing and growth in symbolic processing as well as the observed indirect effect of sequential mediation between non-symbolic processing and later arithmetic may be specific to dyscalculia.

### Arabic Number Processing and Arithmetic

The sequential mediation model presented here also critically extends empirical evidence on the pivotal role of understanding single- and multi-digit Arabic numbers for arithmetic development. As predicted, the ability to process multi-digit numbers was scaffolded onto single-digit number processing. This finding supports the proposed developmental trajectory.

As pinpointed previously (Nuerk et al., 2015), being able to deal with single-digit numbers is an important prerequisite, but perhaps not sufficient for multi-digit number processing. Understanding the relation between decade and unit position is one of the additional steps required for two-digit number processing. It is interesting that in the current study the direct contribution of non-symbolic processing to two-digit number processing missed significance (*p* = 0.061). Future research should test the hypothesis that the ability to represent non-symbolic magnitudes facilitates the acquisition of place-value understanding.

The finding that in our model single-digit processing at T2 (middle of Grade 2) did not show a direct influence on arithmetic growth from T1 to T4 is probably due to the fact that we controlled for differences in symbolic processing at the onset of the study period. This means that the single-digit processing variable actually only reflects changes in task performance through a period of about 6 months. Variance in this variable was predicted by non-symbolic processing skills half a year earlier and in turn contributed significantly to processing of two-digit numbers. Its contribution to arithmetic growth from Grade 2 to Grade 4 was, however, indirect as a mediator of non-symbolic processing.

Although a strong association between performance in single- and two-digit comparison tasks was found in the present study (see also Brankaer et al., 2017), these tasks appear to measure distinct constructs and contribute differently to the prediction of arithmetic performance. Future studies on the development of arithmetic should therefore ideally include both single- and multi-digit number processing tasks. Only few studies have so far dealt with the development of multi-digit number processing (Nuerk et al., 2015). Given the increasing evidence on the high relevance of understanding place-value and multi-digit syntax for arithmetic development (Moeller et al., 2009; Moura et al., 2013; Göbel et al., 2014a), it will be important to investigate the particular challenges children are facing when acquiring complex Arabic numbers and their verbal counterparts. These challenges are mathematical (place-value) as well as linguistic (e.g., inversion of 10s and units in German and other languages) and seem to constitute an important milestone in arithmetic development.

### Limitations

There has been an ongoing discussion on how to best assess non-symbolic magnitude processing skills (Price et al., 2012; Schneider et al., 2017). In the present design, we prioritized having similar tasks for non-symbolic and symbolic processing in order to rule out any confounding effects of differences in task format. Other measures that have been claimed to be more sensitive (e.g., Weber fraction), might have produced stronger effects in our mediation model than the combined measure of accuracy and speed introduced here (including a potential direct effect on later arithmetic).

Similarly, it has been claimed that tasks specifically tapping into cardinal or ordinal number knowledge might be better mediators between non-symbolic processing and mathematical skills. We consider it highly plausible that development of counting plays a crucial role in the mapping of number words and Arabic digits onto non-symbolic processing skills (van Marle et al., 2014). However, although we covered a relatively long developmental period in our longitudinal design, our data do not address the very early foundational processes of this mapping process.

We would also like to remind readers that the current sample was not randomly selected: at the onset of the study period, half of the participants were selected based on below-average performance in arithmetic development. It turned out that later on the majority of children displayed typical arithmetic performance. Still, the distribution of numerical processing and arithmetic skills may not correspond to the general population and replication with more representative samples is advisable.

## Conclusion

In summary, the evidence presented in our study reveals a significant role of non-symbolic magnitude processing in the development of arithmetic during primary school: we could demonstrate that non-symbolic processing skills impacted on growth of arithmetic skills by facilitating the acquisition of symbolic number processing. This evidence indicates that non-symbolic processing should be included as one of the foundational skills in theoretical models of mathematical development. It will also be important to further specify developmental trajectories within the domain of symbolic numerical processing, by, for instance, differentiating between simple processing of one-digit numbers and more complex processes involved in multi-digit processing.

As the indirect effect exerted by non-symbolic processing was small, it seems unlikely that interventions exclusively targeting non-symbolic processing would show satisfactory effects on children’s arithmetic development (Szücs and Myers, 2017). However, drill-practicing number knowledge without providing children with sufficient opportunities to understand how numbers represent non-symbolic magnitudes may be equally inefficient. Training programs should thus focus on understanding and efficiently processing symbolic representation of number, which entails to map them on their inborn non-symbolic representational system (e.g., Kuhn and Holling, 2014; Rauscher et al., 2016). Improving our knowledge of longitudinal developmental trajectories and neurocognitive mechanisms of symbolic and arithmetic processing skills is necessary in order to further advance our understanding of the components that should be integrated in evidence-based tailored intervention programs.

## Data Availability Statement

The dataset analyzed for this study can be found on the Open Sciene Framework at https://osf.io/dfbqj/.

## Ethics Statement

The study was conducted in accordance with the ethical principles of the World Medical Association Declaration of Helsinki. Legal guardians of the participating children gave their written informed consent before data collection.

## Author Contributions

KL developed the study concept and study design and is responsible for data collection. SF performed the data analysis and interpretation under the supervision of HF. SF drafted the manuscript. HF and KL provided critical revisions. All authors approved the final version of the manuscript for submission.

## Conflict of Interest

The authors declare that the research was conducted in the absence of any commercial or financial relationships that could be construed as a potential conflict of interest.
